# PbMYB120 Negatively Regulates Anthocyanin Accumulation in Pear

**DOI:** 10.3390/ijms21041528

**Published:** 2020-02-24

**Authors:** Linyan Song, Xiaoli Wang, Wei Han, Yingying Qu, Zhigang Wang, Rui Zhai, Chengquan Yang, Fengwang Ma, Lingfei Xu

**Affiliations:** 1 College of Horticulture, Northwest A&F University, Yangling 712100, China; 2 State Key Laboratory of Crop Stress Biology for Arid Areas, Northwest A&F University, Yangling 712100, China

**Keywords:** subgroup 4 MYBs, anthocyanin, pear, PbMYB120, repressor

## Abstract

Subgroup 4 R2R3 MYBs play vital roles in the regulation of anthocyanin biosynthesis. However, there is limited knowledge regarding the functions of MYB repressors in pear (*Pyrus × bretschneideri*). Here, PbMYB120 was identified as a potential regulator of anthocyanin biosynthesis. A phylogenetic analysis revealed that PbMYB120 was clustered into the FaMYB1-like clade of the subgroup 4 R2R3 MYBs. *PbMYB120* was expressed higher in red peels than in green peels in five pear cultivars. *PbMYB120* expression was positively correlated with anthocyanin accumulation. However, the transient overexpression of *PbMYB120* led to the inhibition of anthocyanin accumulation and *PbUFGT1* expression. Promoter binding and activation assays indicated that PbMYB120 binds to the promoter of *PbUFGT1* and represses the promoter’s activity. Thus, the inhibition of anthocyanin accumulation by PbMYB120 may be correlated with the repression of *PbUFGT1*. Furthermore, during anthocyanin induction, the expression levels of anthocyanin activators and *PbMYB120* were upregulated. This study demonstrated that *PbMYB120* was highly expressed in pear tissues having higher anthocyanin accumulations but acted as a repressor in the regulation of anthocyanin accumulation. PbMYB120 may work coordinately with anthocyanin activators and serve as a balancer of anthocyanin accumulation.

## 1. Introduction

Coloration is an important factor of fruit quality. Green, yellow, brown, and red-skinned cultivars exist in pear. Among them, red-skinned cultivars have a greater consumer appeal [1]. Many pigment compounds, including betalains, certain carotenoids, some terpenoids, and anthocyanins, can impart red coloration to plants [2]. Anthocyanins are the main pigments for red coloration in pear [3].

Generally, anthocyanin biosynthesis is influenced by internal developmental signals and external environmental signals conjointly [4]. Thus, anthocyanins are stimulated by autogenous sugar and phytohormone signals [5], as well as by many biotic and abiotic stresses, like UV radiation, cold, drought, low nitrogen, heavy metals, wounding, pest attack, and pathogen invasion [6]. Anthocyanins confer bright red, blue, and purple coloration and have anti-oxidation, anti-radical, and anti-pathogen activities [7,8]. Although anthocyanins are considered as by-products of secondary metabolism, the above functions make them important for plant survival under various environmental stress conditions [9,10]. In addition, anthocyanins are beneficial to human health. High intake of anthocyanin-rich foods contributes to the prevention or alleviation of various diseases, including inflammations, cardiovascular diseases, neurogenic diseases, and cancers [11,12]. Many mechanisms can account for the role of anthocyanin in these diseases, for example, anthocyanin extracts can reduce viability and induce the apoptosis and differentiation of cancer cells, and activation of FK506 binding protein 52 by anthocyanins reduces the hyperphosphorylation of Tau protein aggregation and the risk of developing Alzheimer’s disease [12,13,14].

Anthocyanins are end-products of flavonoid metabolism, which is part of the general phenylpropanoid pathway. Anthocyanin biosynthesis is mediated by a series of enzymes, including phenylalanine ammonia-lyase (PAL), cinnamate 4-hydroxylase (C4H), 4-coumarate coenzyme ligase (4CL), chalcone synthase (CHS), chalcone isomerase (CHI), flavone 3-hydroxylase (F3H), flavonoid 3-hydroxylase (F3′H), dihydroflavonol 4-reductase (DFR), anthocyanidin synthase/ leucoanthocyanidin dioxygenase (ANS/LDOX), and UDP glucose:flavonoid 3-O-glucosyl transferase (UF3GT). In pear, genes encoding these enzymes are regulated by numerous factors, including MYB transcriptional factors (TFs), bHLH TFs, TTG1, MADS-box, AP2/ERFs, B-box protein, and SQUAMOSA PROMOTER BINDING PROTEIN-LIKE (SPL) [15,16,17,18,19,20,21,22,23]. In *Arabidopsis thaliana*, the early biosynthetic genes, including those encoding CHS, CHI, F3H and F3′H, are activated directly by three functionally redundant R2R3 MYB TFs of subgroup 7, MYB11, MYB12, and MYB111, while the late biosynthetic genes (LBGs) of the anthocyanin-specific pathway, including those encoding DFR, ANS/LDOX, and UF3GT, are activated by a ternary MYB-bHLH-WD40 (MBW) transcriptional complex, in which the MYB TFs include MYB75/PRODUCTION OF ANTHOCYANIN PIGMENTATION 1 (PAP1), MYB90/PAP2, MYB113, and MYB114 of subgroup 6 [24,25].

MYB TFs play essential roles in the transcriptional regulation of anthocyanin biosynthesis, and other factors also regulate anthocyanin accumulation by regulating or interacting with the MYB TFs [17,26,27,28]. To date, many R2R3 MYB members that function in the transcriptional activation of anthocyanin biosynthetic genes have been identified in pear (*Pyrus × bretschneideri*), including MYB10 and MYB10b/MYB10.1/MYB114 of subgroup 6, and MYB9 of subgroup 5 [16,22,23].

The R2R3 MYBs of subgroup 4 are also involved in the regulation of the phenylpropanoid pathway, including anthocyanin metabolism [29,30]. However, there are limited reports on the roles of subgroup 4 R2R3 MYBs in pear, except for PbMYB3 [23]. A transcriptome analysis of flower buds of the ‘Zaosu’ pear and its red bud sport mutant, ‘Red Zaosu’ pear, revealed that the expression levels of another subgroup 4 R2R3 MYB member (Gene ID was Pbr038870.1) differed significantly between the two cultivars. This gene was named as *PbMYB120* by Li et al. [31]. In this study, the potential function of PbMYB120 in anthocyanin regulation was investigated. By analyzing five pear cultivars that exhibit partially red-coloration of their fruit peels, *PbMYB120* was found to be highly expressed in tissues that contain higher anthocyanin accumulations. However, its transient overexpression in completely faded ‘Red Bartlett’ fruit indicated a negative role of PbMYB120 in anthocyanin accumulation. To elucidate the mechanism of PbMYB120, its role was analyzed during the increase and decrease in anthocyanin accumulation.

## 2. Results

### 2.1. PbMYB120 Belongs to the FaMYB1-Like Clade of the Subgroup 4 R2R3 MYBs

**Figure 1:**
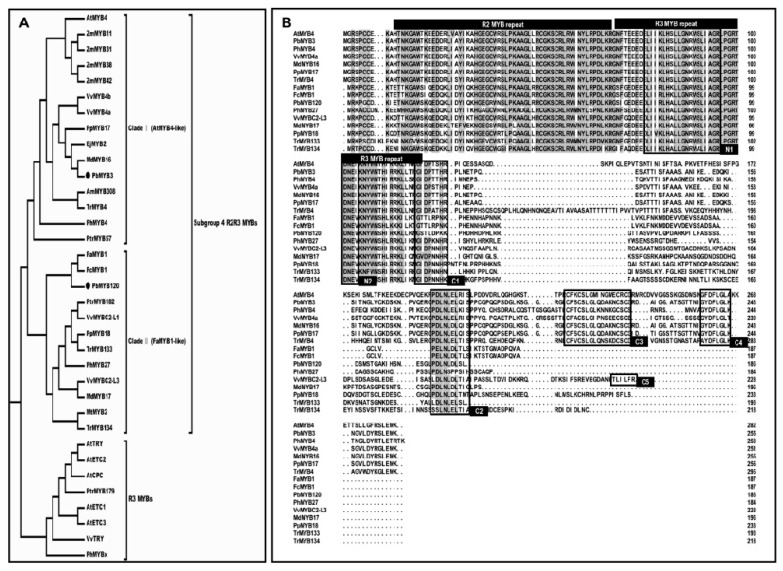
Characterization of PbMYB120. (**A**) Phylogenetic analyses of the subgroup 4 R2R3 MYBs. The subgroup 4 R2R3 MYBs were classified into two subclades: Clade Ⅰ (AtMYB4-like) and Clade Ⅱ (FaMYB1-like). R3 MYBs were added as the out-group. PbMYB3 and PbMYB120 are marked with solid circles. Protein sequences of the subgroup 4 R2R3 MYBs and R3 MYBs used were obtained from the NCBI database. Protein accessions are listed in the “Materials and Methods”. (**B**) Amino acid sequence alignment of typical subgroup 4 R2R3s and the identification of conserved domain motifs. The identified conserved domain motifs are marked with text boxes and wireframes. N1 domain (bHLH-binding domain): [D/E]Lx2[R/K]x3Lx6Lx3R; N2 domain: Characteristic sequence signatures of different clades, with DNEI in the AtMYB4-like repressors and DNEV in the FaMYB1-like repressors; C1 domain: LIsrGIDPxT/SHRxI/L; C2 domain (the EAR repression motif): pdLNLD/ELxiG/S; the C1 and C2 domains are characteristic features of the subgroup 4 R2R3 MYB repressors; C3 domain (zinc finger-like motif): CX2CX9CXC; C4 domain (GY/FDFLGL motif): contributes to the interaction with the Sensitive to ABA and Drought 2 (SAD2); C5 domain: TLLLFR-type repressor motif, which was identified in AtMYBL2 and some FaMYB1-like repressors.

The full-length coding sequence (CDS) of *PbMYB120* was isolated from ‘Red Zaosu’ pear, and no variations were detected at the nucleotide level between the cloned CDS and the reference CDS from the genomic database of ‘Dangshansu’ pear. A phylogenetic analysis of the subgroup 4 R2R3-MYBs revealed that PbMYB120 and PbMYB3 were separately clustered into the FaMYB1-like and AtMYB4-like clades, respectively (Figure 1A). The identification of conserved domains showed that PbMYB120 and PbMYB3 both contain the characteristic C1 and C2 (also called the ethylene-responsive element binding factor-associated amphiphilic repression motif, ‘the EAR motif’ for short) domains of the subgroup 4 R2R3-MYBs (Figure 1B). Additionally, the N1 domain associated with bHLH interaction and the N2 domain associated with classification of the subgroup 4 R2R3-MYBs were also identified in both PbMYB120 and PbMYB3 (Figure 1B).

### 2.2. PbMYB120’s Expression Pattern Positively Correlated with Anthocyanin Accumulation

In many plant species, subgroup 4 R2R3 MYBs of the FaMYB1-like clade are involved in the regulation of the flavonoid pathway, including the anthocyanin branch [29,30]. Because PbMYB120 showed high homology to FaMYB1, it was considered as a potential regulator of anthocyanin biosynthesis (Figure 1A).

**Figure 2:**
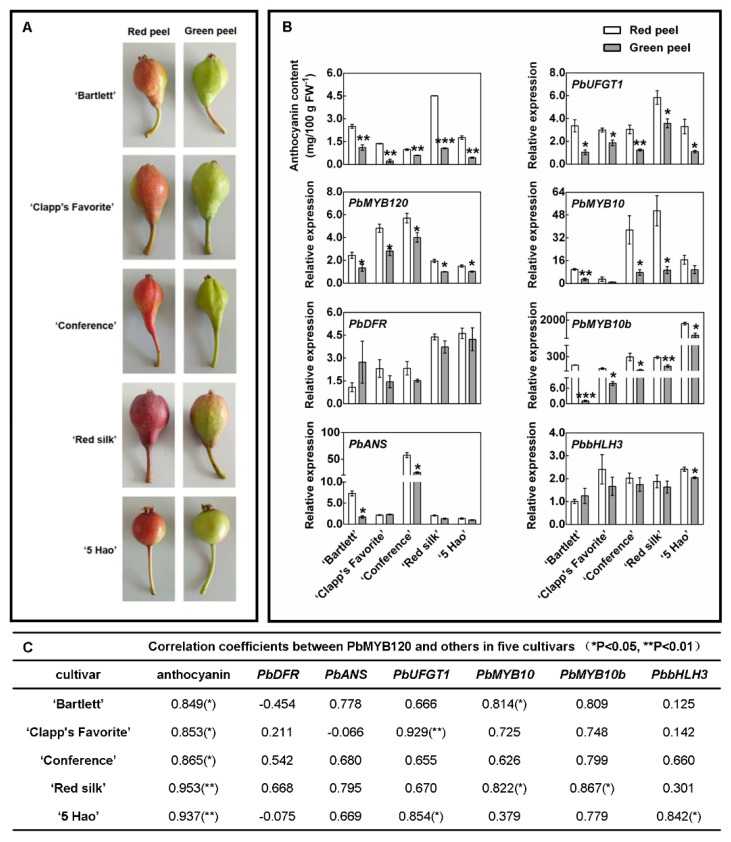
Expression analyses of *PbMYB120* in fruit of five pear cultivars having uneven coloration. (**A**) Fruit coloration of five pear cultivars, ‘Bartlett’, ‘Clap’s Favorite’, ‘Conference’, ‘Red silk’ and ‘5 Hao’. (**B**) Analyses of anthocyanin contents and expression levels of *PbMYB120* and anthocyanin biosynthesis-related genes in the red and green peels of the five tested pear cultivars. Data are the means ± standard errors (SEs) of three biological replicates. Asterisks indicate significant differences as assessed by Student’s *t* test: * *p* < 0.05, ** *p* < 0.01, *** *p* < 0.001. (**C**) Correlation analyses between the expression of *PbMYB120* and both anthocyanin accumulation and expressions of anthocyanin biosynthesis-related genes. Data represent Pearson’s correlation coefficients that were calculated using SPSS 13.0. * *p* < 0.05, ** *p* < 0.01.

To determine whether *PbMYB120* was involved in anthocyanin regulation, its expression level was detected in fruit of five pear cultivars having uneven color distributions. Among them, ‘Bartlett’, ‘Clapp’s Favorite’, ‘Conference’ and ‘Red Silk’ pears are red colored on the side exposed to the sun but green colored on the shaded side. The coloration of the ‘5 Hao’ pear is irregular. It gradually turns from green to fully red, with only a portion of the fruit showing a similar coloring pattern as the above cultivars (Figure 2A).

The anthocyanin contents were higher in the red-colored portions than in the green-colored portions of fruit from all five cultivars. Similarly, *PbMYB120* was highly expressed in red-colored portions having higher anthocyanin accumulations (Figure 2B). An expression analysis of anthocyanin-related LBGs and regulatory genes showed that *PbUFGT1* and *PbMYB10b* were also expressed higher in the red peels than in the green peels of all five cultivars. However, *PbANS*, *PbMYB10*, and *PbbHLH3* were expressed higher in the red peels than in the green peels of one or some of the five cultivars (Figure 2B). A correlation analysis demonstrated that *PbMYB120* expression was positively correlated with the accumulation of anthocyanin and the expression of anthocyanin-related genes (Figure 2C).

### 2.3. PbMYB120 Behaves as a Repressor of Anthocyanin Biosynthesis

**Figure 3:**
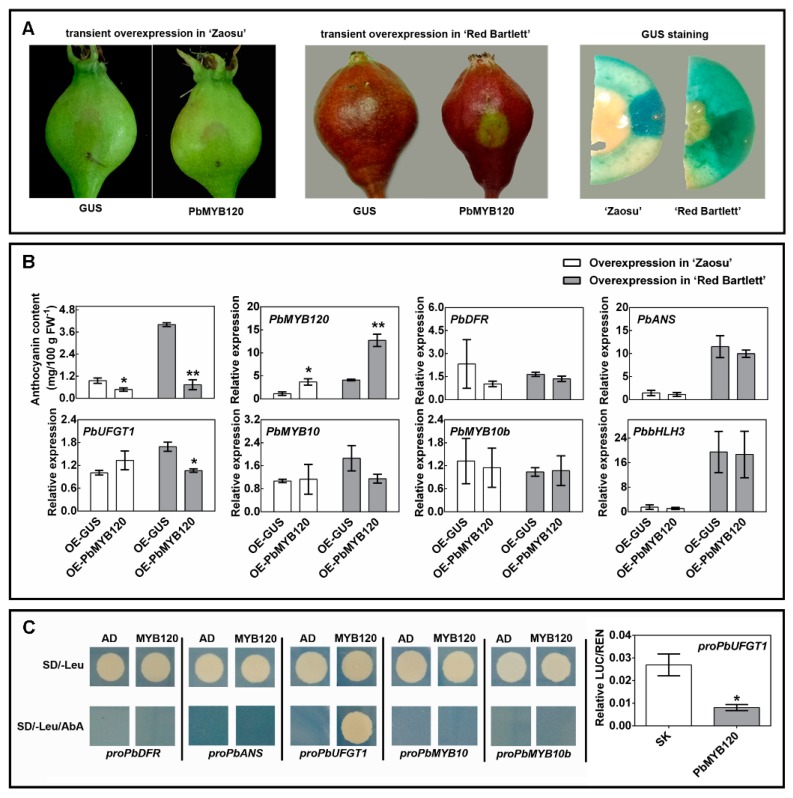
Transient overexpression and protein interaction analysis of PbMYB120. (**A**) Phenotypes of ‘Zaosu’ and ‘Red Bartlett’ fruit transiently overexpressing *PbMYB120* and the determination of the transfection efficiency as assessed by GUS staining. (**B**) Analysis of anthocyanin biosynthesis and *PbMYB120* expression in ‘Zaosu’ and ‘Red Bartlett’ fruit transiently overexpressing *PbMYB120*. (**C**) Promoter-binding analyses of anthocyanin late biosynthetic genes (LBGs) and regulatory genes as assessed by Y1H assays, and a *PbUFGT1* promoter activation analysis as assessed by the dual-luciferase reporter assay. Data are the means ± SEs of three biological replicates. Asterisks indicate significant differences as assessed by Student’s *t* test: * *p* < 0.05, ** *p* < 0.01.

Transient transformation assays have been used for quick confirmation of the potential gene functions in pear [17,22,23]. To determine whether PbMYB120 could induce anthocyanin biosynthesis, a transient *PbMYB120*-overexpression assay was carried out in young fruit of the ‘Zaosu’ pear. However, no obvious red coloration was induced by the transient overexpression of *PbMYB120*, as expected (Figure 3A). Instead, anthocyanin accumulation was inhibited slightly by *PbMYB120*′s transient overexpression. Nonetheless, no changes were found in the expression levels of anthocyanin-related LBGs and regulatory genes (Figure 3B).

The EAR motif is a predominant transcription repression motif in plants [32]. Many TFs containing the EAR motif function as repressors of the biological processes that they coordinate [33]. This promoted us to hypothesize that PbMYB120 putatively functions as a negative regulator of anthocyanin biosynthesis.

To verify this hypothesis, a transient *PbMYB120*-overexpression assay was carried out in the completely faded ‘Red Bartlett’ pear. As shown in Figure 3A,B, *PbMYB120′*s overexpression delayed coloration and repressed anthocyanin accumulation. A subsequent expression analysis of anthocyanin-related LBGs and regulatory genes revealed that only the expression level of *PbUFGT1* was inhibited in *PbMYB120*-overexpression fruit (Figure 3B). Furthermore, a yeast one-hybrid assay showed that PbMYB120 could bind to the promoter of *PbUFGT1*, but not to the promoters of *PbDFR*, *PbANS*, *PbMYB10*, and *PbMYB10b*, and this was in accordance with the gene expression analyses (Figure 3B,C). The *PbUFGT1* promoter activation assay using the dual-luciferase reporter assay system indicated that PbMYB120 could repress the promoter activity of *PbUFGT1* (Figure 3C). These data suggest that PbMYB120 represses anthocyanin biosynthesis directly through the repression of *PbUFGT1*.

### 2.4. PbMYB120 Is Expressed in an Anthocyanin-Dependent Manner and Forms a Negative Feedback Loop Regulating Anthocyanin Accumulation

The EAR motif containing repressors can suppress gene expression under non-inductive conditions, and they are induced under inductive conditions to control gene activation and prevent potential damage from run-away responses [33]. Thus, we speculate that PbMYB120 may behave as a balancer that functions opposite to activators, to optimize the anthocyanin content in pear. To test this hypothesis, the role of PbMYB120 during the increase and decrease of anthocyanin accumulation was analyzed.

**Figure 4:**
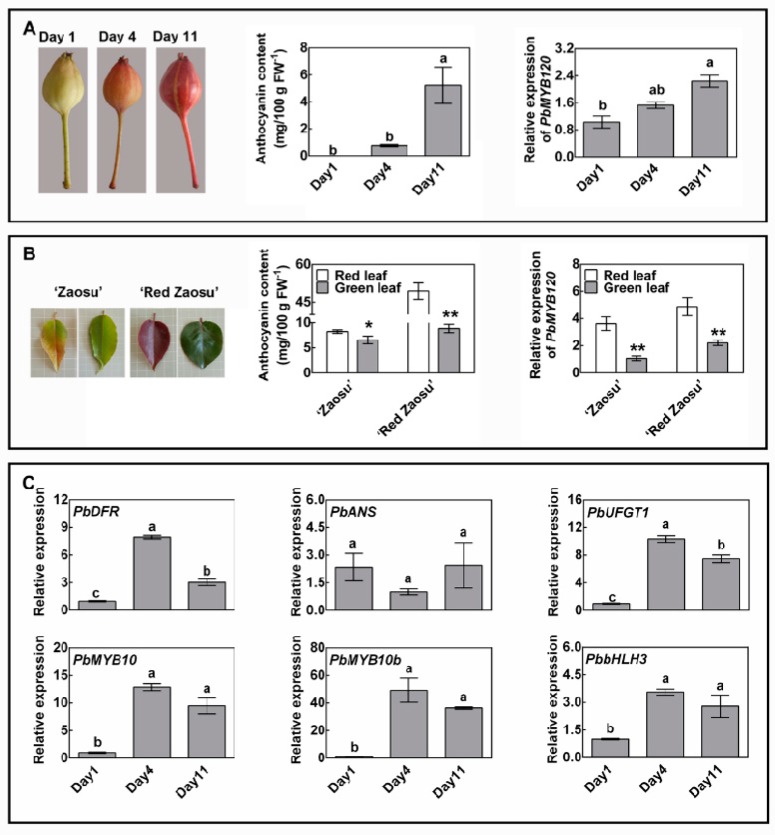
Role of PbMYB120 in increase and decrease of anthocyanin accumulation. (**A**) Analysis of anthocyanin accumulation and *PbMYB120* expression during light-induced fruit coloration of ‘Red Zaosu’. Young fruit of ‘Red Zaosu’ were bagged and faded completely after 30 days of shading. Days 1, 4, and 11 represent 1, 4, and 11 days after removing the fruit bags, respectively. (**B**) Analyses of anthocyanin accumulation and *PbMYB120* expression during the natural fading of leaf coloration of ‘Zaosu’ and ‘Red Zaosu’. Young leaves with red coloration and mature leaves with green coloration were analyzed. Leaves were light- or deep-red colored at the young stages. The red coloration decreased during the leaf developmental process and disappeared eventually. Leaves were green colored at the mature stages. (**C**) Expression analysis of genes involved in anthocyanin pathway in light-induced fruit coloration of ‘Red Zaosu’. Data are the means ± SEs of three biological replicates. Differences between two samples were assessed using Student’s *t* test at a 95% confidence level and are indicated by asterisks (* *p* < 0.05, ** *p* < 0.01). Differences among the three samples were assessed using one-way ANOVA with Tukey’s honestly significant difference test at a 95% confidence level and are indicated by lowercase letters above the error bar.

During the light-induced fruit coloration, *PbMYB120* was up-regulated as the anthocyanin accumulation increased (Figure 4A). Similarly, during the natural color fading of leaf, *PbMYB120* was down-regulated as the anthocyanin accumulation decreased (Figure 4B). These results imply that *PbMYB120* is expressed in an anthocyanin-dependent manner. Because of the negative regulation of PbMYB120 in anthocyanin biosynthesis, we speculated that it may form a negative feedback loop regulating anthocyanin accumulation. In addition, anthocyanin LBGs *PbDFR* and *PbUFGT1*, and regulatory genes *PbMYB10*, *PbMYB10b*, and *PbbHLH3*, were also up-regulated after light exposure but did not continue to increase during the later stages. This might occur to avoid excessive anthocyanin accumulation, and possibly resulted from the direct repression of *PbUFGT1* and the indirect repression of the MBW complex by PbMYB120 (Figure 4C).

## 3. Discussion

### 3.1. Roles of the Subgroup 4 R2R3 MYBs in the Regulation of Anthocyanin in Pear

Among the numerous regulators, MYB TFs, especially R2R3-type MYB TFs, play dominant roles in the phenylpropanoid pathway. Functions of the R2R3 MYB activators in the regulation of different branches of the phenylpropanoid pathway have been well studied. After the report of the regulation of the phenylpropanoid pathway by subgroup 4 R2R3 MYB repressors in *Antirrhinum majus* [34], *Arabidopsis thaliana* [35], and *Fragaria* × *ananassa* [36], attention has been focused on the regulation of phenolic compounds by R2R3 MYB repressors.

The subgroup 4 R2R3 MYBs have been phylogenetically classified into the AtMYB4-like and FaMYB1-like clades [29,37]. The AtMYB4-like clade’s members are mainly involved in the regulation of the general phenylpropanoid pathway and the lignin-branched pathway, while the FaMYB1-like clade’s members are mainly involved in the regulation of the flavonoid-branched pathway [29,30,37].

Transient RNAi of an AtMYB4-like member, PbMYB3, represses the biosynthesis of flavonols and anthocyanins derived from the flavonoid-branched pathway [23]. In this study, a FaMYB1-like member, PbMYB120, repressed anthocyanin accumulation in pear, and this inhibition might have resulted partially from repression of *PbUFGT1* (Figure 3).

### 3.2. Divergent Functions of MYB Activators and Broad Effects of MYB Repressors in the Regulation of Different Branches of the Flavonoid Pathway

The opposing effects of MYB activators and MYB repressors on anthocyanin biosynthesis have been reported in different plant species. Most of the MYB activators are involved in the regulation of special flavonoids. For example, in grape (*Vitis vinifera*), VvMYBA1 and VvMYBA2 are involved in the regulation of anthocyanins, VvMYBPA1 and VvMYBPA2 are involved in the regulation of proanthocyanidins, and VvMYBF1 is involved in the regulation of flavonols [38,39]. However, no functional differentiation among MYB repressors has been reported in the regulation of different branches of flavonoids. They have a broad range of effects on flavonoid pathway regulation. For example, VvMYBC2-L1 and VvMYBC2-L3 repress both anthocyanins and proanthocyanidins [30,37]. Because the target gene of PbMYB120, *PbUFGT1*, encodes an enzyme that catalyzes the glycosylation of anthocyanins and flavonols, it was inferred that PbMYB120 also represses other flavonoid compounds, like flavonols [40].

### 3.3. Mechanisms of Subgroup 4 R2R3 MYB Repressors in Anthocyanin Regulation

MYB activators promote anthocyanin biosynthesis through the activation of LBGs [24]. However, a complex mechanism is involved in the negative regulation of anthocyanin biosynthesis by MYB repressors [29,37]. Reviews on the anthocyanin biosynthesis suggest that FaMYB1-like anthocyanin repressors may generally exert their functions in indirect ways by interfering with the interaction and assembly of MYB and bHLH members in the MBW activation complex, and thus repress the transcriptional activity of the MBW activation complex [29,30]. Some AtMYB4-like repressors can also repress anthocyanin biosynthesis, and they generally exert their functions in direct ways. For example, apple (*Malus × domestica*) MYB16 directly binds *MdANS* and *MdUFGT*, and inhibits their expression through the C-terminal EAR motif. AtMYB3 inhibits the biosynthesis of sinapoyl malate and anthocyanin directly by targeting and repressing *AtC4H* [29,41,42].

In this study, the FaMYB1-like anthocyanin repressor, PbMYB120, targeted the promoter of *PbUFGT1* and repressed its activity (Figure 3C). This indicated that PbMYB120 could inhibit anthocyanin biosynthesis, at least in a direct way. The expression of *UFGT* has been inhibited in anthocyanin MYB repressor-overexpression lines. However, only a few MYB repressors, apple MdMYB16 and peach PpMYB18, for instance, can target the promoter of *UFGT* or inhibit the promoter activity of *UFGT* directly [30,41,42]. The actions of lignin and flavonol MYB regulators usually do not require the bHLH cofactor, while the actions of anthocyanin and proanthocyanidin MYB regulators generally do require the bHLH cofactor [30]. Thus, PbMYB120 might also inhibit anthocyanin biosynthesis in an indirect way by competing with PbMYB10 and PbMYB10b to bind the PbbHLH3 cofactor. Further studies are required to prove this hypothesis.

### 3.4. MYB Activators and Repressors Act Cooperatively in the Regulation of Anthocyanin Accumulation

Anthocyanin accumulation is regulated by both positive and negative regulators [43]. The expression patterns of anthocyanin activators *PbMYB10* and *PbMYB10b* are consistent with the accumulations of anthocyanin in many pear cultivars [21,44]. In this study, the expression pattern of *PbMYB120* was also consistent with the accumulation of anthocyanin in many pear cultivars (Figure 2 and Figure 4). This indicated that both MYB activators and repressors are involved in the regulation of anthocyanin biosynthesis in pear.

Anthocyanin accumulation increases in response to various biotic and abiotic stresses to prevent plants from stress-related damage and improve survival rates [6,9,10]. Meanwhile, anthocyanin activators and repressors are also induced or inhibited after short- or long-term stress treatments [45]. Light exposure also induces anthocyanin accumulation [18,46,47]. In this study, the MYB activators *PbMYB10* and *PbMYB10b* and the MYB repressor *PbMYB120* were all induced after light exposure. The up-regulation of *PbMYB120* and retention of anthocyanin activators at the late stages of light-induced anthocyanin accumulation might contribute to the optimization of the anthocyanin content in pear (Figure 4). This could be explained by MYB repressors being induced under inductive conditions to control gene activation and prevent potential damage from possible run-away responses [33]. This explanation is supported by anthocyanin MYB repressors being activated by anthocyanin MYB activators, as demonstrated by overexpression analyses of MYB activators and promoter activation analyses of MYB repressors [48,49,50,51,52].

## 4. Materials and Methods

### 4.1. Plant Materials

All the pear samples used were obtained from a pear orchard in Meixian, Shaanxi, China. For gene cloning, pericarps of ‘Red Zaosu’ were collected at 80 days after flower bloom (DAFB). For gene expression analysis of *PbMYB120* and correlation analysis with anthocyanin biosynthesis, five pear cultivars, ‘Bartlett’, ‘Clapp’s Favorite’, ‘Conference’, ‘Red Silk’ and ‘5 Hao’ that have different colorations on different sides of their fruit were sampled at 40 DAFB. In addition, fruit peels with red and green coloration were taken separately. For transient overexpression, young fruit of ‘Zaosu’ at 20 DAFB were directly used for agroinfiltration, while those of ‘Red Bartlett’ were bagged at 10 DAFB, and the fruit bags were removed after the color had completely faded. For the anthocyanin accumulation-induction analysis, fruit of ‘Red Zaosu’ at 10 DAFB were shaded using fruit bags, and the bags were removed after 30 days of shading before exposure to natural light. Then, fruit were sampled at 1, 4, and 11 days after removing the fruit bags. For anthocyanin accumulation-reduction analysis, young and mature leaves of ‘Zaosu’ and ‘Red Zaosu’ pear were sampled at 10 DAFB. For all the samples, fruit peels and leaves were sampled and immediately frozen in liquid nitrogen and stored at −80 °C for DNA and total RNA isolation and anthocyanin extraction. Three biological repeats were performed for all the assays, and eight fruit and six leaves were collected for each biological repeat.

### 4.2. Cloning and Sequence Analysis of PbMYB120

The full-length CDS of *PbMYB120* was isolated from ‘Red Zaosu’ pear. Amino acid sequences of PbMYB120, PbMYB3, and subgroup 4 MYBs of other plants were aligned using ClustalW. The phylogenetic tree was constructed using MEGA5.10 with the neighbor-joining statistical method. In addition, 1000 bootstrap replications were performed for testing of phylogeny. R3-type MYB repressors were added as the out-group. The AtMYB4-like and FaMYB1-like clades were searched for previously reported conserved domain motifs of the subgroup 4 MYBs [37].

The protein accessions used were as follows: PbMYB3 (*Pyrus* × *bretschneideri*, ALU57828); AtMYB4 (*Arabidopsis thaliana*, AAC83582), AtTRY (AED96321), AtCPC (AEC10691), AtETC1 (AEE27280), AtETC2 (AEC08385), and AtETC3 (OAO97552); ZmMYB11 (*Zea mays*, AIB05021), ZmMYB31 (CAJ42202), ZmMYB38 (AIB04526), and ZmMYB42 (CAJ42204); MtMYB2 (*Medicago truncatula*, AES99346); PtrMYB57 (*Populus trichocarpa*, PNS24054), PtrMYB182 (AJI76863), and PtrMYB179 (AJI76864); AmMYB308 (*Antirrhinum majus*, P81393); PhMYB4 (*Petunia* × *hybrida*, ADX33331), PhMYB27 (AHX24372), and PhMYBx (AHX24371); FaMYB1 (*Fragaria* × *ananassa*, AAK84064); FcMYB1 (*Fragaria chiloensis*, ADK56163); VvMYB4a (*Vitis vinifera*, ABL61515), VvMYB4b (ACN94269), VvMYBC2-L1 (ABW34393), VvMYBC2-L3 (AIP98385), and VvTRY (ABW34395); MdMYB16 (*Malus domestica*, ADL36756) and MdMYB17 (ADL36757); PpMYB17 (*Prunus persica*, ALO81020) and PpMYB18 (ALO81021); EjMYB2 (*Eriobotrya japonica*, AID56314); and TrMYB4 (*Trifolium repens*, AMB27079), TrMYB133 (AMB27081), and TrMYB134 (AMB27082).

### 4.3. Expression Analyses by Quantitative Real-Time PCR

Total RNA was isolated using an RNAprep Pure Plant Kit (DP441, TIANGEN, Beijing, China) and quantified using a Nanodrop 2000 spectrophotometer (Thermo Fisher Scientific Inc., Wilmington, DE, USA). The RNA quality was evaluated by agarose gel electrophoresis. Then, 1 µg of total RNA was reverse-transcribed to cDNA using the PrimeScript RT Reagent Kit with gDNA Eraser (RR047A, TaKaRa, Dalian, China).

Quantitative real-time PCR (qRT-PCR) was performed on a StepOnePlus™ Real-Time PCR System (Applied Biosystems, Thermo Fisher Scientific, Albany, NY, USA) using the TB Green Dye (RR820A, TaKaRa). Primers used for qRT-PCR are listed in Table S1. Every qRT-PCR was performed in three biological replicates, and *PbACTIN* (GenBank NM_001302286.1) was used as the reference gene for normalization of the templates. Expression profiles were analyzed using the ΔΔ*C*T algorithm [53]. In each chart, gene expressions were normalized using the sample that showed the lowest gene expression (or highest ΔCT value). Especially, for gene expression data used in the subsequent correlation analysis in each cultivar (Figure 2C), the gene with the highest ΔCT was used for normalization, regardless of the highest ΔCT occurring in red peel or green peel.

### 4.4. Extraction and Determination of Anthocyanin Content

Total anthocyanin was extracted, measured, and calculated using the pH difference method, as modified by Wang et al. [54] with slight modifications. The extractions and measurements used were four times smaller in volume than in the original system. The final anthocyanin concentration was expressed as mg/100 g fresh weight^−1^ (mg/100 g FW^−1^).

### 4.5. Correlation Analyses between PbMYB120 Expression and Anthocyanin Content

Bivariate correlations were carried out between *PbMYB120* expression and the anthocyanin contents using SPSS 20 with Pearson’s method. In addition, correlations between *PbMYB120* expression and expression levels of anthocyanin-related LBGs and activators were also analyzed.

### 4.6. Transient Overexpression Assays Using Agrobacterium Injections of Pear Fruit

Transient functional verification was conducted as described by Zhai et al. [23]. The full-length CDS of *PbMYB120* and the β-glucuronidase gene (*GUS*) were amplified from ‘Red Zaosu’ and pBI121, respectively. They were then inserted individually into the multiple cloning site (MCS) of pGreen II 0029-62SK to generate constructs expressing *35S::PbMYB120* and *35S::GUS*, respectively. The infusion vectors were transferred into *Agrobacterium tumefaciens* strain EHA105 containing the pSoup helper. The agrobacterium strains were incubated in Luria–Bertani medium, collected, and suspended in the infiltration buffer (10 mM MgCl_2_, 10 mM MES, pH 5.6, and 150 μM acetosyringone) with gentle shaking for 2–4 h. Final OD600 values of the agrobacterium strains were adjusted to 0.3 prior to use in plant infiltration. The transient overexpression of *35S::GUS* was used as the control check of the overexpression assay. Pericarp at the injection site was peeled and quick-frozen in liquid nitrogen for gene expression and anthocyanin analyses. To test the transfection efficiency, histochemical staining for GUS activity in trans-overexpression fruit was performed as described previously [55].

### 4.7. Yeast One-Hybrid Assay

Yeast one-hybrid assay was conducted using the Matchmaker^®^ Gold Yeast One-Hybrid System (Clontech, Mountain View, CA, USA). The full-length CDS of *PbMYB120* and 800–1500 bp promoters of *PbDFR*, *PbANS*, *PbUFGT1*, *PbMYB10*, and *PbMYB10b* were amplified and inserted into the MCS of Y1H prey vector pGADT7 AD and bait vector pAbAi, respectively. The bait constructs were linearized with BstBI or BbsI and transformed into yeast strain Y1HGOLD to generate the bait yeast strains. Then, the bait yeast strains were cultured on SD/–Ura medium containing 100–200 ng ml^–1^ of aureobasidin A (AbA) to select its minimum inhibitory concentration. The prey plasmids were transformed independently into the bait yeast strains and cultured on SD/–Leu medium containing the minimum inhibitory concentration of AbA to test for interactions.

### 4.8. Dual Luciferase Assay

To confirm the results of yeast one-hybrid, a dual luciferase assay was performed as previously described [55,56]. The promoter of *PbUFGT1* was inserted into the MCS of the reporter vector pGreen II 0800 LUC to generate a construct expressing *proPbUFGT1::LUC*. The effector vector expressing *35S::PbMYB120* was the same as that used in the transient overexpression assay. Agrobacterium cultures containing the reporter and effector were prepared as described above and mixed at a ratio of 1:1. Mixed suspension cultures were injected into the abaxial sides of the leaves of six-leaved *Nicotiana benthamiana* seedlings. The tobacco plants were grown in a light growth chamber with 16 h of daylight. At 2 days after infiltration, leaves were harvested, and firefly luciferase and Renilla luciferase were measured using the dual luciferase assay reagents (Promega, Madison, WI, USA) and an Infinite M200pro multifunctional microplate detector. Data were expressed as firefly: Renilla luciferase activity ratios. Six independent repeats were performed.

### 4.9. Statistical Analysis

The statistical significances of differences were calculated using Student’s *t*-test and one-way ANOVA with Tukey’s honestly significant difference test at a 95% confidence level. Figures were drawn using GraphPad Prism 6.01 (GraphPad Prism, San Diego, CA, USA) software.

## 5. Conclusions

**Figure 5:**
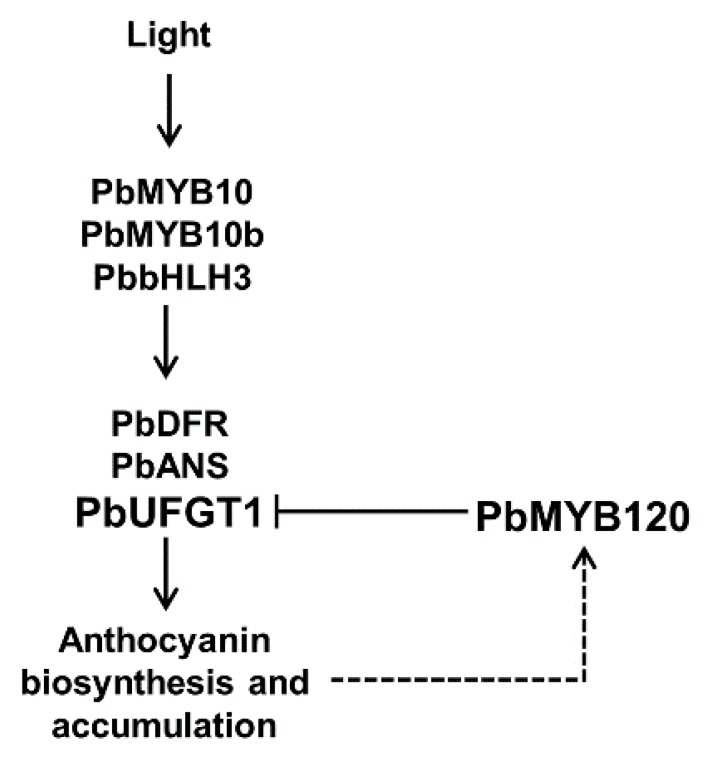
Predicted mechanism of PbMYB120 in the regulation of anthocyanin accumulation. PbMYB120 inhibited anthocyanin biosynthesis by the direct repression of *PbUFGT1*. *PbMYB120* was expressed in an anthocyanin-dependent manner. *PbMYB120′*s expression may be induced by light, anthocyanin activators, or increased anthocyanin accumulation. It formed a negative feedback loop regulating anthocyanin accumulation by the direct repression of *PbUFGT1* and possible indirect interference with the MYB-bHLH-WD40 (MBW) complex.

In this study, a potential FaMYB1-like subgroup 4 R2R3 MYB, PbMYB120, was identified as a negative regulator of anthocyanin accumulation. A predicted working model of PbMYB120 was proposed and presented in Figure 5. PbMYB120 negatively regulated anthocyanin biosynthesis in a direct way through the repression of *PbUFGT1*. Additionally, *PbMYB120* was expressed in an anthocyanin-dependent manner. During the light-induced anthocyanin biosynthesis, *PbMYB120* and anthocyanin activators were all up-regulated, suggesting that PbMYB120 serves to balance anthocyanin accumulation through a negative regulatory feedback loop.

## Supplementary Materials

Supplementary materials can be found at https://www.mdpi.com/1422-0067/21/4/1528/s1.
