# Supplementary material for: PbMYB120 Negatively Regulates Anthocyanin Accumulation in Pear
Source: Int J Mol Sci. 2020 Feb 24;21(4):1528. doi: 10.3390/ijms21041528 (PMC7073189; doi:10.3390/ijms21041528)
Supplement: Supplementary file 1 [file ijms-21-01528-s001.pdf]

Table S1. Primers used for gene cloning, relative expression analysis and vector construction in this study.

Primer Name	Primer Sequence (5′-3′)	Purpose
PbMYB120-F	ATGAGGAAGCCTGGTTGTGAT	CDS cloning
PbMYB120-R	CTAGAGTGAGAGGTCGAGATTCAAG	CDS cloning
PbActin-qF	TGAGTCACACTGTGCCAATCTATG	qRT-PCR
PbActin-qR	TGGTGAACATGTACCCTCTTTCAG	qRT-PCR
PbMYB120-qF	GGGAAGGACAGACAATGAGGTG	qRT-PCR
PbMYB120-qR	CACCAGTACTCATGGAATCCGAC	qRT-PCR
PbDFR-qF	GCCCAAAGTTGTCCAATGTCT	qRT-PCR
PbDFR-qR	GCTTGTAAGTGAATCCCAAATCTG	qRT-PCR
PbANS-qF	CTGGGCAGCTTGAGTGGGAG	qRT-PCR
PbANS-qR	CCACCAACTTCTTTCTCCAGCC	qRT-PCR
PbUFGT1-qF	GTGGAGGACGTGTTGGAGATAGG	qRT-PCR
PbUFGT1-qR	CCTCGCACGATGCTTTATTGG	qRT-PCR
PbMYB10-qF	AGGGCTGCATGTCCCAGC	qRT-PCR
PbMYB10-qR	CATGCCACATTTACAAGCAAGG	qRT-PCR
PbMYB10b-qF	ATACAGACAATGTTGATGGAACACC	qRT-PCR
PbMYB10b-qR	CGATTGCTTGAGACTTTGGACC	qRT-PCR
PbbHLH3-qF	TAATCGAGAGTGATGGGCTGTTG	qRT-PCR
PbbHLH3-qR	TCTTGCCACTCACGTTATCCTTC	qRT-PCR
62SK (GUS)-F	tagaactagtggatccATGTTACGTCCTGTAGAAACCCCA	transient overexpression
62SK (GUS)-R	cggtatcgataagcttTCATTGTTTGCCTCCCTGCT	transient overexpression
62SK (PbMYB120)-F	tagaactagtggatccATGAGGAAGCCTGGTTGTGAT	transient overexpression & dual-luciferase assay
62SK (PbMYB120)-R	cggtatcgataagcttCTAGAGTGAGAGGTCGAGATTCAAG	transient overexpression & dual-luciferase assay
pAbAi (PbDFR)-F	agcttgaattcgagctcGATCTTTTCTGGATCATTTTTG	Y1H assay
pAbAi (PbDFR)-R	atgcctcgaggtcgacAACGGATTCGGACTCGGA	Y1H assay
pAbAi (PbANS)-F	agcttgaattcgagctcATCCCAGATATGTTTACTGCTCA	Y1H assay
pAbAi (PbANS)-R	atgcctcgaggtcgacCACCATTTTTGGAGCTGGCT	Y1H assay
pAbAi (PbUFGT1)-F	agcttgaattcgagctcACTTAGGTGGATCCTAGTCATT	Y1H assay
pAbAi (PbUFGT1)-R	atgcctcgaggtcgacCGGCGGTGCCATTACAAC	Y1H assay
pAbAi (PbMYB10)-F	agcttgaattcgagctcCTGATAGCGTGGTCTCCTGCAAG	Y1H assay
pAbAi (PbMYB10)-R	atgcctcgaggtcgacGCTTATCTTTTGCCTGCTACCCAC	Y1H assay
pAbAi (PbMYB10b)-F	agcttgaattcgagctcTGCAATGCGACCGTAAATCG	Y1H assay

pAbAi (PbMYB10b)-R	atgcctcgaggtcgacGATCATAAGGCCACCGACGTG	Y1H assay
AD (PbMYB120)-F	gccatggaggccagtgaattcATGAGGAAGCCTGGTTGTGAT	Y1H assay
AD (PbMYB120)-R	cagctcgagctcgatggatccCTAGAGTGAGAGGTCGAGATTCAAG	Y1H assay
0800 Luc (PbUFGT1)-F	cggtatcgataagcttGTGCCTCCCGTAGGGCTAC	dual-luciferase assay
0800 Luc (PbUFGT1)-R	tagaactagtggatccCGGCGGTGCCATTACAAC	dual-luciferase assay
